# Three-dimensional dental microwear in type-Maastrichtian mosasaur teeth (Reptilia, Squamata)

**DOI:** 10.1038/s41598-023-42369-7

**Published:** 2023-11-09

**Authors:** Femke M. Holwerda, Jordan Bestwick, Mark A. Purnell, John W. M. Jagt, Anne S. Schulp

**Affiliations:** 1 Royal Tyrrell Museum of Palaeontology, P.O. Box 7500, Drumheller, AB T0J 0Y0 Canada; 2 Department of Geosciences, Utrecht University, Princetonlaan 8a, 3584 CB Utrecht, The Netherlands; 3 School of Geography, Geology and the Environment, Centre for Palaeobiology Research, University of Leicester, Leicester, LE1 7RH UK; 4 School of Geography, Earth and Environmental Sciences, University of Birmingham, Edgbaston, Birmingham, B15 2TT UK; 5 Natuurhistorisch Museum Maastricht, De Bosquetplein 6-7, 6211 KJ Maastricht, The Netherlands; 6 Naturalis Biodiversity Center, P.O. Box 9517, 2300 RA Leiden, The Netherlands

**Keywords:** Palaeoecology, Palaeontology, Behavioural ecology

## Abstract

Mosasaurs (Squamata, Mosasauridae) were large aquatic reptiles from the Late Cretaceous that filled a range of ecological niches within marine ecosystems. The type-Maastrichtian strata (68–66 Ma) of the Netherlands and Belgium preserve remains of five species that seemed to have performed different ecological roles (carnivores, piscivores, durophages). However, many interpretations of mosasaur diet and niche partitioning are based on qualitative types of evidence that are difficult to test explicitly. Here, we apply three-dimensional dental microwear texture analysis (DMTA) to provide quantitative dietary constraints for type-Maastrichtian mosasaurs, and to assess levels of niche partitioning between taxa. DMTA indicates that these mosasaurs did not exhibit neatly defined diets or strict dietary partitioning. Instead, we identify three broad groups: (i) mosasaurs *Carinodens belgicus* and *Plioplatecarpus marshi* plotting in the space of modern reptiles that are predominantly piscivorous and/or consume harder invertebrate prey, (ii) *Prognathodon saturator* and *Prognathodon sectorius* overlapping with extant reptiles that consume larger amounts of softer invertebrate prey items, and (iii) *Mosasaurus hoffmanni* spanning a larger plot area in terms of dietary constraints. The clear divide between the aforementioned first two groups in texture-dietary space indicates that, despite our small sample sizes, this method shows the potential of DMTA to test hypotheses and provide quantitative constraints on mosasaur diets and ecological roles.

## Introduction

Mosasaurid reptiles were large, predominantly marine squamates, and one of only a few groups of squamates that became fully aquatic^1–4^. During the final c. 30 million years of the Cretaceous, mosasaurs rapidly evolved a wide array of morphologies, and occupied a range of ecological niches in marine ecosystems around the globe^1,2,5,6^. By the end of the Cretaceous they were firmly established as a group, often hypothesized as apex predators in most ecosystems they inhabited^1^.

**Figure 1:**
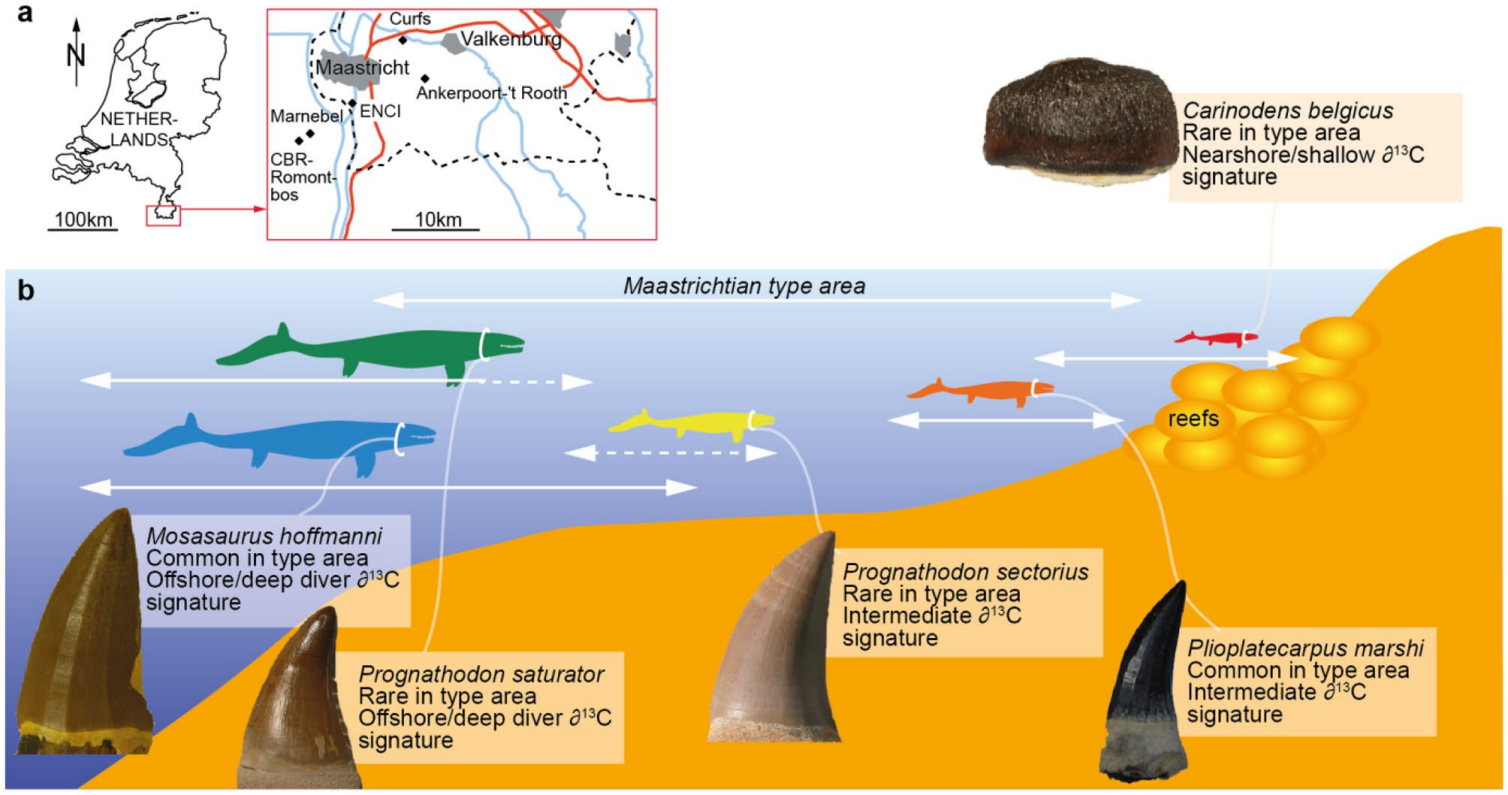
(**a**) Type-Maastrichtian area in the southeast Netherlands and northeast Belgium with the most important quarries indicated (diamonds within inset). (**b**) The five mosasaur taxa known from the Type Maastrichtian include *Mosasaurus hoffmanni*, *Prognathodon saturator*, *Prognathodon sectorius*, *Plioplatecarpus marshi* and *Carinodens belgicus*. Placement of the taxa along a nearshore-offshore gradient follows the ∂^13^C interpretations in Ref.^10^. Mosasaur silhouettes and colour coding retained from Refs.^9,10^ for consistency. Tooth images not to scale (see “Materials and methods” section for sources).

The type-Maastrichtian biocalcarenites (68–66 Ma) of the southeastern part of the Netherlands and northeastern Belgium^7^ (Fig. 1) are relatively rich in mosasaur remains. Currently, five mosasaur taxa are recognized from these strata: the large (c. > 10 m long) *Mosasaurus hoffmanni* and *Prognathodon saturator*; the intermediately-sized *Prognathodon* cf. *sectorius* (see notes in “Materials and methods” section) and *Plioplatecarpus marshi*; and the diminutive (c. 3 m long) *Carinodens belgicus*^8,9^ (Fig. 1). The mosasaur fossils are from the uppermost part of the Gulpen Formation (Lanaye Member) and from the entire Maastricht Formation (Valkenburg, Gronsveld, Schiepersberg, Emael, Nekum and Meerssen members)^7,10^ (Fig. 1). The general trend is for units to reflect biocalcarenite deposition in increasingly shallower subtropical settings^7,11^.

Mosasaur ecological niche occupancy has been reconstructed through several direct or indirect lines of evidence. These include: tooth morphology^12,13^; biomechanical experiments^14^; range of visual foraging via orbit size (e.g.,^15,16^; content fossils (i.e., fossilized stomach and throat contents and coprolites), e.g.,^17–20^; and stable isotope signatures (e.g.^9,21^). However, each type of evidence has associated problems that limit the robustness of mosasaur dietary hypotheses. Gut content is informative but, to date, has not been found with any type-Maastrichtian mosasaurs, and their paucity across the whole mosasaur fossil record hampers reliable extrapolations from other taxa^22^. Furthermore, fossilized gut contents generally only provide dietary ‘snapshots’, i.e., items consumed within the last few hours or days before an organism’s death. These can thus be biased by the retention of indigestible items^23–25^. Stable isotope analyses can inform on possible foraging habitats but they generally provide only coarse indications of the relative trophic levels of taxa^26–28^.

One robust approach to test hypotheses of diet and dietary partitioning in mosasaurs involves dental microwear; the microscopic wear patterns on tooth surfaces, formed by tooth-food contact and/or tooth-tooth occlusion (e.g.^29–37^). So far, the only microwear analysis performed on the type-Maastrichtian mosasaur fauna has focused on two-dimensional (2D) wear patterns (i.e., visual identification of features such as scratches and pits from scanning electron microscopy; SEM) in the presumably durophagous *Carinodens belgicus*^33^. However, 2D approaches have well-known limitations arising particularly from inter-observer error in identifying wear features and the difficulties of standardizing surface illumination when generating images^30,38–41^. Three-dimensional dental microwear texture analysis (DMTA), in contrast, quantifies the sub-micrometre scale texture characteristics on tooth surfaces through scale-senstive fractal analysis^38,39^ or ISO-derived parameters^42,43^, using the results to reconstruct the diets of species and/or populations. This technique does not rely on assumptions of direct relationships between the morphology and inferred functions of teeth^44,45^. Although originally developed for use in terrestrial mammals, recent work has demonstrated that dietary signals can be recovered from the non-occlusal tooth surfaces of extant reptiles, which do not chew their food in the same way, allowing discrimination between dietary guilds even when sample sizes are low^36,46^. These data have subsequently served as multivariate frameworks for testing and constraining dietary hypotheses of extinct reptiles^34,35,46^. DMTA has also been applied to a variety of aquatic vertebrates^28,43,45,47,48^.

**Figure 2:**
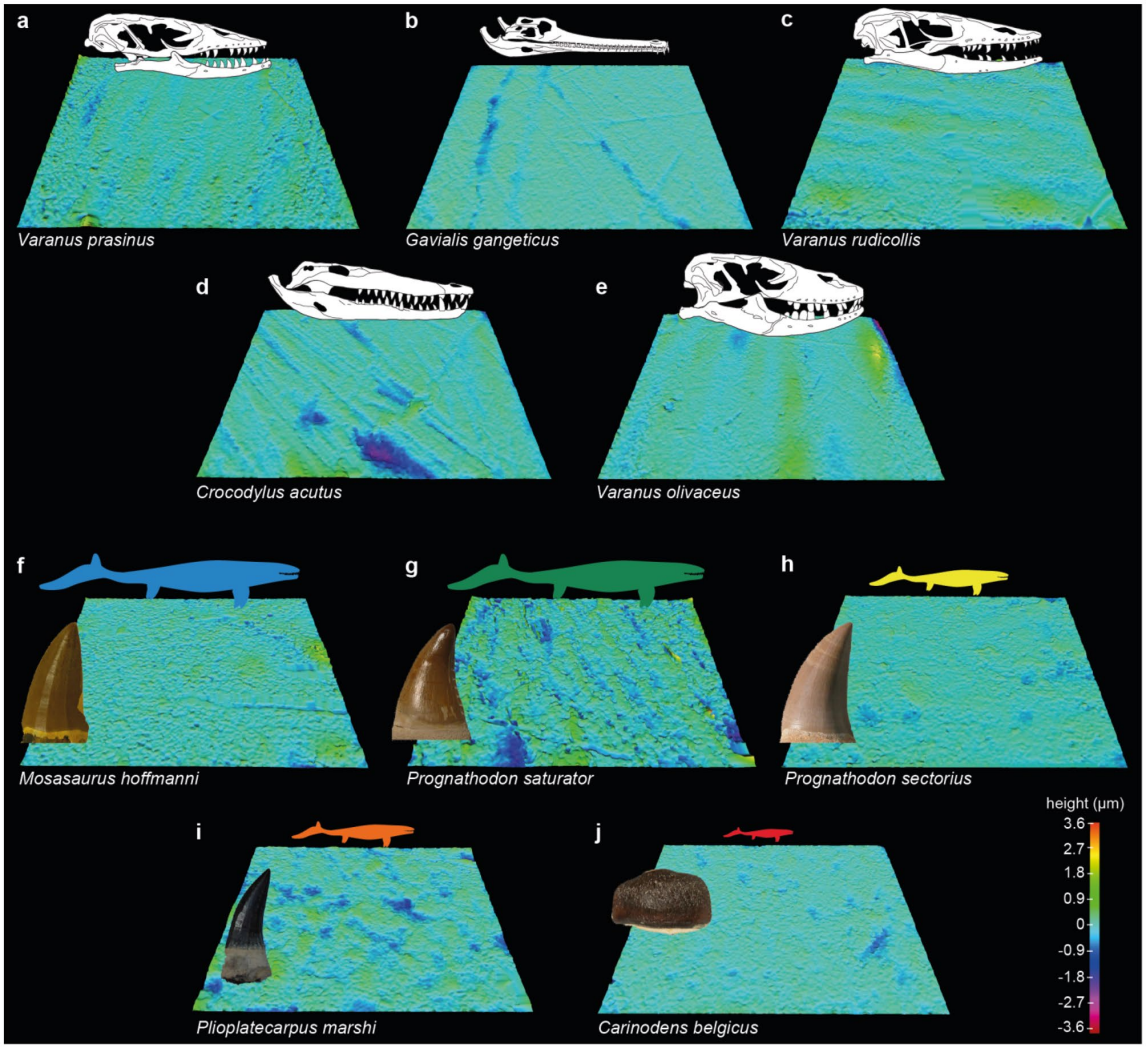
Example scale-limited three-dimensional tooth surfaces of extant reptile dietary guilds and mosasaurs. (**a**–**e**) Reptile dietary guilds; (**a**) ‘softer’ invertebrate consumer (*Varanus prasinus*; emerald tree monitor), (**b**) piscivore (*Gavialis gangeticus*; gharial), (**c**) carnivore (*Varanus rudicollis*; roughneck monitor lizard), (**d**) ‘harder’ invertebrate consumer (*Crocodylus acutus*; American crocodile) and (**e**) omnivore (*Varanus olivaceus*; Gray’s monitor lizard). (**f**–**j**) Mosasaurs; (**f**) *Mosasaurus hoffmanni* (principal component (PC) plot number 5 in Fig. 3), (**g**) *Prognathodon saturator* (PC number 14), (**h**) *Prognathodon sectorius* (PC number 17), (**i**) *Plioplatecarpus marshi* (PCA number 11) and (**j**) *Carinodens belgicus* (PC number 1). Measured areas 146 × 110 µm in size. Topographic scale in micrometres. Extant reptile skull diagrams and mosasaur tooth images not to scale (see “Materials and methods” section for sources). Extant reptile tooth surfaces and skull diagrams of *G. gangeticus*, *C. acutus* and *V. olivaceus* adapted from Bestwick et al.^35,36^, by Anne Schulp under a Creative Commons Attribution 4.0 International License https://creativecommons.org/licenses/by/4.0/ to include the *V. prasinus* and *V. rudicollis* diagrams and the mosasaur tooth images and surface textures.

Here, we conduct the first exploratory analysis of DMTA in large marine reptiles, applying it to well-preserved isolated tooth crowns of all five mosasaur taxa known from the type area for the Maastrichtian. We used the multivariate framework of Bestwick et al.^35,36^ comprising of an existing dataset of microwear texture data from extant varanid lizards and crocodilians with known diets^36^. Figure 2a–j shows example digital elevation models of scale limited surfaces of teeth from mosasaurs and extant reptile guilds. Although mosasaurs and crocodilians are very distantly related (clades Squamata and Archosauria, respectively^2,49^), previous DMTA of reptiles has shown that microwear texture differences are more strongly linked to diet than to phylogeny^36,46^. The crocodilians included in our analysis exhibit diets that are both similar as well as different to the varanids and thus produce a robust framework. Furthermore, previous analyses have demonstrated the potential for DMTA to provide dietary information in aquatic vertebrates^28,43,45,48^.

## Results

Our analyses use the multivariate texture-dietary spaces developed by Bestwick et al.^34,36^ based on extant varanid lizards and crocodilians with known diets, which are assigned to dietary guilds. Four texture parameters differ between guilds, and our analysis projects mosasaurs into the principal component analysis (PCA) based on these parameters. The parameters are: Spk, the mean height of peaks above the top of the core, with higher values indicating a surface composed of high peaks; Sds, the number of summits per unit area of the surface, with higher values indicating that peaks make up a greater proportion of the surface; Vmp, the volume of material contained within peaks that make up the highest 10% of the surface; and Smr1, the percentage of the surface that is composed of peaks that are higher than the top of the core. Several of these parameters, and many others, are derived from the areal material ratio curve: a cumulative density function derived from the scale-limited tooth surface by plotting the distribution of height values for a surface as a cumulative percentage. The peaks, valleys and core material of tooth surfaces are defined on the basis of this curve, with the core for material ratio parameters equivalent to the volume that lies between the heights of the surface delimited by the extrapolated intercept of the minimum slope of the curve. Bestwick et al. (Ref.^36^, Table S2, Fig. S2) provide more details and a graphical explanation based on dental microwear texture data.

**Figure 3:**
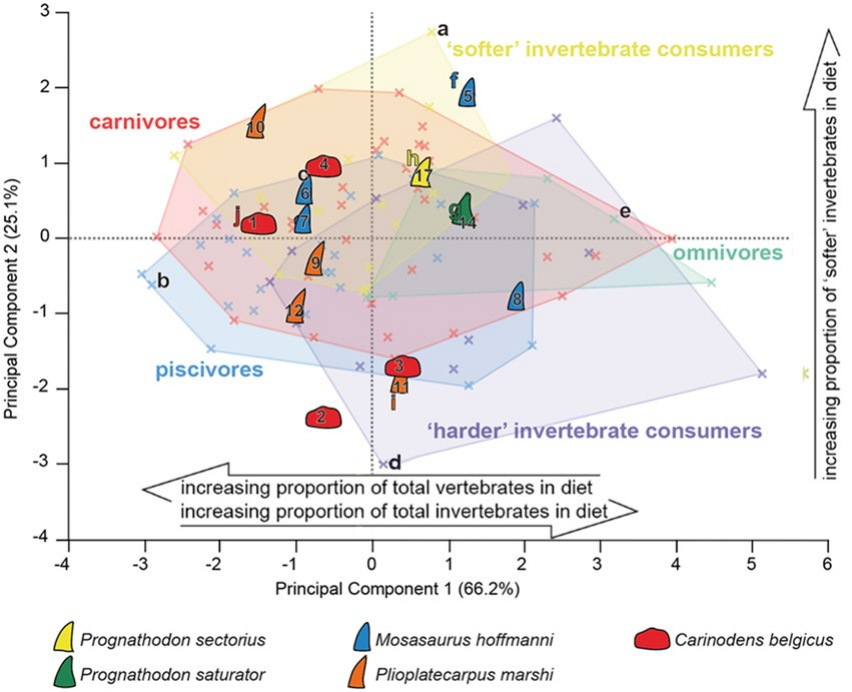
Principal component textural analysis of three-dimensional microwear textures in extant reptiles and mosasaurs. Texture-dietary space of four International Organization for Standardization (ISO 25178-2) texture parameters (Spk, Sds, Vmp, Smr1) for extant reptiles and mosasaurs. Texture-dietary space based on extant reptile data with mosasaurs projected onto the first two axes as unknown datum points. Extant and fossil specimens with associated letters represent surfaces (**a–j**) in Figure. Arrows show significant correlations of dietary characteristics along PC axes 1 and 2. Mosasaur specimen information can be found in Table S1. Mosasaur tooth silhouettes not to scale (see “Materials and methods” section for sources). Texture-dietary space adapted from Bestwick et al.^35^ by Jordan Bestwick and Anne Schulp under a Creative Commons Attribution 4.0 International License https://creativecommons.org/licenses/by/4.0/ to include the mosasaur data.

Summarizing the key elements of texture-dietary space defined by principal component axes 1 and 2 (Fig. 3): PC 1 negatively correlates with proportions of total vertebrates in reptile diets and positively with total invertebrates. PC 2 positively correlates with proportions of ‘softer’ invertebrates (see^36^, Table S3 for all dietary correlation results). Analysis of variance (ANOVA) of the PCA finds that PCs 1 and 2 differ significantly between dietary guilds in extant reptiles (PC 1, *F* = 4.9316, d.f. = 4, 90, *P* = 0.0012; PC 2, *F* = 4.6676, d.f. = 4, 90, *P* = 0.0018). Piscivores differ from ‘harder’ invertebrate consumers and omnivores (PC 1, Tukey HSD); ‘harder’ invertebrate consumers differ from carnivores and ‘softer’ invertebrate consumers; ‘softer’ invertebrate consumers differ from piscivores (PC 2, Tukey HSD).

Projecting the type-Maastrichtian mosasaur data into this texture-dietary space reveals that most specimens fall within the bounds of the extant reptiles, with only two specimens, one of *Carinodens belgicus* and one of *Mosasaurus hoffmanni* (specimen numbers 2 and 5, respectively) falling outside of the texture-dietary space (Fig. 3). *Prognathodon saturator* and *Prognathodon sectorius* are known from a single specimen of each taxon; Fig. 3 shows only a single tooth from each taxon, but we collected data from other teeth of the same individuals, and these cluster closer together in multivariate space than the teeth of the other taxa, which represent single teeth from multiple individuals (see Supplementary Fig. 1).

The two smaller, but morphologically dissimilar mosasaurs *Carinodens belgicus* and *Plioplatecarpus marshi* occupy very similar areas towards the left of the texture-dietary space, with values of less than zero for PC 1 and more dispersed PC 2 values, reflecting generally less rough surface textures (see^36^). The differences between these two clusters of mosasaurs are statistically significant; PC 1 values for *Prognathodon* are significantly higher than the *Carinodens*-*Plioplatecarpus* group (*t* =  − 3.69, *P* = 0.0015; one-tailed test), as are the PC 2 values (*t* =  − 1.82, *P* = 0.047; one-tailed test). This indicates tooth surfaces made up of more and higher peaks, containing a greater proportion of the material of the surface compared to tooth surfaces with more negative values for PC1.

*Mosasaurus hoffmanni* is the most broadly distributed species along PCs 1 and 2, with centroids of the area covered by these specimens overlapping with all those of the extant dietary guilds.

## Discussion

DMTA of extant reptiles provides a multivariate space defined by microwear textures that have dietary significance^36,46^. This supports the hypothesis that the distribution of mosasaur species, when plotted in this space, and their similarities and significant differences in dental microwear texture, can be interpreted in terms of diet.

However, several factors limit the validity of our interpretations, such as our small sample size for the mosasaurs, their morphological differences to extant reptiles, and their completely aquatic habitat. Moreover, the degree of overlap between extant reptiles assigned to different dietary categories limits the confidence that can be placed in direct comparisons between the diets of the extant reptiles and the mosasaurs in this exploratory study.

Nevertheless, our dataset shows some clear differences between the *Carinodens*-*Plioplatecarpus* group, which plot toward the left of the texture-dietary space, generally with PC 1 values < 0, and the *Prognathodon saturator* and *Prognathodon sectorius* group, plotting in the top right quadrant. This tentatively suggests niche partitioning, although more specimens of *Prognathodon* are needed to confirm this.

Overall, the areas of occupied texture-dietary space suggests that the type-Maastrichtian mosasaurs exhibit a similar dietary space to the extant reptiles, i.e., they consumed food/prey items of a comparable range of material properties. The amount of overlap with multiple guilds occupied by extant reptiles suggests a degree of dietary generalism exhibited by all mosasaurs, but the separation between species groups indicates some level of dietary specialisation and partitioning. This allows us to assess the likelihood of niche partitioning between taxa, to test dietary hypotheses, and to discuss the implications on our understanding of Late Cretaceous marine ecosystems.

The separation of the *Prognathodon* species group from the *Carinodens*–*Plioplatecarpus* group in the texture-dietary spaces provides strong evidence that these species consumed different food items, which in turn is in favour of niche partitioning. Interestingly, this result is similar to that from analyses of carbon isotope ratios in which *Prognathodon* species were more similar to each other than to *Carinodens* and *Plioplatecarpus*, and vice versa^9^. DMTA suggests that the diet of *Prognathodon* species, in terms of material properties, was more similar to reptiles that consume higher proportions of invertebrates and, more specifically, higher proportions of softer invertebrates. The distribution of *Prognathodon* samples most strongly overlaps with extant softer invertebrate consumers and omnivores, the latter guild, like *Prognathodon*, falling around the PC texture-dietary space with positive PC 1 values. Carnivory, however, cannot be ruled out, as the *Prognathodon* samples lie close to the areas of the texture-dietary spaces with the highest density of carnivore samples. From their distribution in the diet-texture spaces, it is unlikely that *Prognathodon* consumed significant amounts of harder prey or fish. These findings partially contrast with previous dietary interpretations of *Prognathodon*. *Prognathodon sectorius*, for example, has been interpreted as a mixed consumer of fish and squid based on its tooth morphology^50^, while *Prognathodon saturator* has been interpreted as a carnivore and a generalist based on its tooth morphology and robust mandibular morphology^6,19,51,52^. The possibility that *Prognathodon* microwear textures have been disproportionately influenced by consumption of vertebrates with ‘harder’ body parts, such as the shells of turtles—a commonly suggested food item for *Prognathodon saturator*^9,19,51^—cannot be ruled out. Nevertheless, and despite the previously mentioned small sample size, our results provide new, yet tentative, dietary constraints for type-Maastrichtian mosasaurs.

The diet of the *Carinodens*-*Plioplatecarpus* group is likely to have included both fish and prey with relatively harder exteriors. Similar to this mosasaur group, most extant piscivorous reptiles have negative PC 1 values, and the mosasaur samples with low and negative PC 2 values fall close to the area of the texture-dietary spaces with the highest density of reptiles from the harder invertebrate consumers. The general picture of negative PC 1 values for *Carinodens* and *Plioplatecarpus* is consistent with the trend in the reptile data towards consumption of more vertebrates with increasingly negative PC 1 values. Our results partially corroborate previous dietary interpretations of *Plioplatecarpus* as primarily a piscivore based on its tooth morphology^9,50^. In contrast, our results differ from previous interpretations of *Carinodens* as an obligate durophage based on multiple lines of evidence, including: tooth morphology^53^; visual identification of tooth wear features (i.e., scratches and pits^33^) and biomechanical investigations into possible feeding behaviours^14^. This highlights the potential for using DMTA as an independent technique for constraining mosasaur diets and understanding levels of niche partitioning.

The relatively large areas occupied by *Mosasaurus* within the texture-dietary spaces, and overlap with multiple extant guilds within each space, suggests this mosasaur was more of a dietary generalist, with perhaps slight preferences for tetrapods and invertebrates. This corroborates previous interpretations of *Mosasaurus* as an apex predator that consumed whatever it wished, based on its tooth morphology, overall size and associations with other marine reptiles^9,53^.

Despite our small sample sizes, our results provide novel insights into the structure of the type-Maastrichtian marine ecosystem and, more broadly, exemplifies the applicability of DMTA to mosasaurs. The detection of three relatively distinct dietary groups indicates that the ecosystem was healthy and productive enough to support five species of large (> 3 m long) predatory reptiles^9,10^. However, the lack of complete separation between these groups in the texture-dietary spaces, and the large areas occupied within these spaces by some species, indicates that dietary competition did occur between mosasaurs. This interpretation is supported by extant co-occurring aquatic reptiles that also exhibit minimal dietary partitioning, such as the spectacled caiman (*Caiman crocodilus*) and black caiman (*Melanosuchus niger*) in the Amazon River^54^, and the Siamese crocodile (*Crocodylus siamensis*) and false gharial (*Tomistoma schlegelii*) in Indonesia^55^. Several non-mutually exclusive factors could explain how the type-Maastrichtian ecosystem supported mosasaur dietary competition. First, dietary generalism and opportunism could have reduced both inter- and intra-specific competition (particularly for *Mosasaurus*), as exhibited by extant varanids^56,57^ and some crocodilians^27,58^. Second, size-based resource partitioning may have occurred. For example, although *Plioplatecarpus* and *Carinodens* comprise a similar dietary group, the former is nearly twice as large as the latter^9^ and is therefore unlikely to have fed on exactly the same food items. Third, differences in habitat usage and foraging behaviours would have created a degree of spatial partitioning. For example, carbon isotope signatures of type-Maastrichtian mosasaurs suggest that larger species, such as *Mosasaurus* and *Prognathodon saturator*, are more likely to have fed in open waters, with more and deeper diving in their foraging, while smaller species such as *Carinodens* fed in shallower waters closer to the shore^9,21^.

Analysis of additional tooth samples will hopefully generate more robust dietary ranges of mosasaurs and subsequently enable likely or unlikely mechanisms of dietary partitioning to be established in a representative way.

## Conclusions

We used DMTA to provide quantitative constraints on the diets of type-Maastrichtian mosasaurs and on degrees of dietary partitioning between these taxa. Our analyses suggest that the type-Maastrichtian mosasaurs did not exhibit neatly defined diets or strict dietary partitioning. Rather, mosasaurs formed several dietary groups comprising one or more species that show small, yet noticeable, preferences for vertebrate and/or invertebrate prey while exhibiting at least some degree of dietary generalism. This contrasts with several hypotheses of mosasaur diets and indicates potentially higher levels of competition than previously appreciated. Our results provide novel insights into the diversity of ecological roles performed by mosasaurs, the structure of the type-Maastrichtian ecosystem and the applicability of DMTA to mosasaurs. Increased sampling will provide further robust constraints on diets and ecological roles of type-Maastrichtian taxa and future application to other mosasaurs will enhance our understanding of the functioning and evolution of Late Cretaceous marine ecosystems.

## Materials and methods

### Material

Three-dimensional tooth microwear textures were sampled from 18 mosasaur tooth crowns, covering the five taxa listed above (*Carinodens belgicus*, *n* = 4; *Mosasaurus hoffmanni*, *n* = 4; *Plioplatecarpus marshi*, *n* = 4. *Prognathodon saturator* and *Prognathodon sectorius* are known only from single specimens from the type Maastrichtian; three teeth from each were initially sampled, and one tooth output per species selected a posteriori (the isolated tooth LV150 is *P*. *sectorius*, but the skeletal specimen, is cf. *sectorius*). DMT data for mosasaurs were compared with the data of Ref.^36^, which sampled six crocodilian and seven monitor lizard species, to serve as an extant multivariate reference framework for mosasaur dietary analysis.

Extant and fossil specimens from the published dataset by Ref.^36^ were sampled from the Field Museum of Natural History, Chicago, USA (FMNH); Grant Museum of Zoology, University College London, UK (LDUCZ); Natuurhistorisch Museum Maastricht, Maastricht, The Netherlands (NHMM); The Natural History Museum, London, UK (NHMUK); University of Oxford Museum of Natural History, Oxford, UK (OUMNH); Teylers Museum, Haarlem, Netherlands (TM); Florida Museum of Natural History, Gainesville, USA (UF); and the National Museum of Natural History, Smithsonian Institute, Washington D.C., USA (USNM). See Table S1 for the complete specimen list. The *Crocodylus acutus*, *Gavialis gangeticus* and *Varanus olivaceus* skull diagrams in Fig. 3 were adapted from Ref.^35^, and the *Varanus prasinus* and *Varanus rudicollis* diagrams were drawn from UF 56949 and UF 63622 respectively. Mosasaur silhouettes and tooth images are adapted from Refs.^9,10^ with the same colour codes used to distinguish between taxa.

Extant reptiles were assigned to one of five dietary guilds that account for the relative ‘intractability’ (roughly equivalent to hardness) of prey as food: carnivores (tetrapod consumers); piscivores (fish consumers); ‘harder’ invertebrate consumers (invertebrates with hard exoskeletons, e.g., beetles, crustaceans and shelled gastropods); ‘softer’ invertebrate consumers (invertebrates with less hard exoskeletons, e.g., crickets, grasshoppers, dragonflies, damselflies and ants); omnivores (combination of plant and organic matter). See^36^ for how each species was assigned to a guild.

### Tooth sampling

Mosasaur specimens were cleaned with acetone-soaked cotton swabs to remove any grease or dirt and consolidant. Microwear data from all extant and extinct reptiles were acquired from non-occlusal (non-chewing) labial surfaces, as close to the tooth apex as possible. Wear facets that likely formed from tooth-tooth occlusion from the opening and closing of jaws, characterized by their vertical orientation, elliptical shape and parallel features^59^, were not sampled. High-fidelity tooth cast replicas were then created using standard laboratory protocols^36,60^. First, negative tooth moulds were produced using President Jet Regular Body polyvinylsiloxane (Coltène/Whaledent Ltd., Burgess Hill, West Sussex UK). Initial moulds taken from each specimen were then discarded to remove any remaining dirt and all analyses were performed on respective second moulds. Casts were then made from these moulds using EpoTek 320 LV Black epoxy resin mixed to manufacturer’s instructions. Resin was cured for 24 h under 200 kPa (2 Bar/30 psi) of pressure (Protima Pressure Tank 10 L) to improve casting quality. Small casts were mounted onto 12.7 mm SEM stubs using President Jet polyvinylsiloxane with the labial, non-occluding surfaces orientated apically to optimise data acquisition. All casts were sputter coated with gold for 3 min (SC650, Bio-Rad, Hercules, CA, USA) to optimize capture of surface texture data. Replicas produced using these methods are statistically indistinguishable from original tooth surfaces^60^.

### Three-dimensional surface texture data acquisition

Surface texture data acquisition follows standard laboratory protocols^36^. Data were captured using an Alicona Infinite Focus microscope G4b (IFM; Alicona GmbH, Graz, Austria; software version 2.1.2), using a × 100 objective lens, producing a field of view of 146 × 100 µm. Lateral and vertical resolution were set at 440 nm and 20 nm, respectively. Casts were orientated so that labial surfaces were perpendicular to the axis of the objective lens.

All 3D data files were processed using Alicona IFM software (version 2.1.2) to remove dirt particles from tooth surfaces and anomalous data points (spikes) by manual deletion. Data were levelled (subtraction of least squares plane) to remove variation caused by differences in tooth surface orientation at the time of data capture. Files were exported as .sur files and imported into Surfstand (software version 5.0.0 Centre for Precision Technologies, University of Huddersfield, West Yorkshire, UK). Scale-limited surfaces were generated through application of a fifth-order robust polynomial to remove gross tooth form and a robust Gaussian filter (wavelength λ_c_ = 0.025 mm)^45,61^. ISO 25178-2 areal texture parameters (International Organization for Standardization, 2012) were then generated from each scale-limited surface. Descriptions of ISO parameters can be found in Table S2 from Ref.^36^.

### Statistical analyses

Log-transformed texture data were used for analyses as some of the texture parameters were non-normally distributed (Shapiro–Wilk, *P* > 0.05). The parameter Ssk was excluded from analysis as it contains negative values and thus cannot be log-transformed. To test the hypothesis that mosasaur microwear texture differences reflect dietary differences, mosasaur microwear data were independently projected as unknown data points onto the axes of a principal components analysis (PCA) and a canonical variates analysis (CVA, a form of linear discriminant analysis) of extant reptile dietary guild microwear differences. All DMTA analyses were performed with JMP Pro 12 (SAS Institute, Cary, NC, USA).

### Supplementary Information

Supplementary Table S1.

Supplementary Legends.

Supplementary Figure 1.

## Data Availability

Museum specimen information, raw microwear texture data and PC values of all sampled extant and extinct reptiles can be found in Table S1.
