# Supplementary material for: Three-dimensional dental microwear in type-Maastrichtian mosasaur teeth (Reptilia, Squamata)
Source: Sci Rep. 2023 Nov 9;13:18720. doi: 10.1038/s41598-023-42369-7 (PMC10636054; doi:10.1038/s41598-023-42369-7)
Supplement: Supplementary file 2 — Supplementary Legends. [file 41598_2023_42369_MOESM2_ESM.docx]

**Supplementary Figure 1**. Principal component analysis of all three-dimensional microwear textures in extant reptiles and mosasaurs. Mosasaur specimens can be found in Table S1. Mosasaur tooth silhouettes not to scale, see Methods for sources. See Figure 3 for other specifications. Texture-dietary space adapted from Bestwick et al., (2020), and by Anne Schulp under a Creative Commons Attribution 4.0 International License to include mosasaur data <https://creativecommons.org/licenses/by/4.0/>
