# Supplementary figures and images for: Three-dimensional dental microwear in type-Maastrichtian mosasaur teeth (Reptilia, Squamata)
Source: Sci Rep. 2023 Nov 9;13:18720. doi: 10.1038/s41598-023-42369-7 (PMC10636054; doi:10.1038/s41598-023-42369-7)

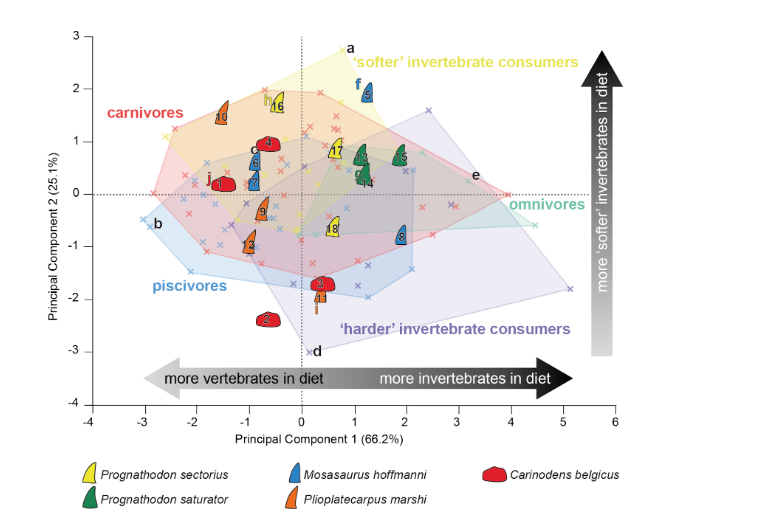

Supplement: Supplementary file 3 — Supplementary Figure 1. [file 41598_2023_42369_MOESM3_ESM.png]
